# 

**Published:** 1786

**Authors:** 


### CATALOGUE OF BOOKS.

1. A TREATISE of Midwifery, theoretical and practical; illuſtrated with copper plates. By *David Spence*, M. D. Licentiate of the Royal College of Phyſicians, and Fellow of

 the the Society of Scottish Antiquaries. 8vo. Edinburgh, 1785.

2. Principles of Midwifery; or, Puerperal Medicine. By *John Aitken*, M. D. Fellow of the Royal College of Surgeons, Member of the Royal Medical, Physical, and Antiquary Societies, one of the Surgeons of the Royal Infirmary, Lecturer of Anatomy, Surgery, and Midwifery, and Honorary President of the Chirurgo-Obstetrical Society of Edinburgh. The third Edition, enlarged, and illustrated with thirty Engravings. 8vo. *Murray*, London, 1785.

3. Observations on the Scurvy; with a Review of the Theories lately advanced on the Disease, and the Opinions of Dr. Milman refuted from Practice. By *Thomas Trotter*, Surgeon of His Majesty's Navy, and Member of the Royal Medical Society of Edinburgh. 8vo. *Robinson*, London, 1785.

4. A Dissertation on Elective Attractions. By Sir *Torbern Bergman*, late Professor of Chemistry at Upsal, and Knight of the Royal Order of Vasa. Translated from the Latin by the Translator of Spallanzani's Dissertations. 8vo. *Murray*, London, 1785.

5. Experiments and Observations relating to various Branches of Natural Philosophy; with a Cona Continuation of Obſervations on Air. By *Joſeph Prieſtley*, LL. D. F. R. S. Vol. III. 8vo. *Johnſon*, London, 1786.

6. Medical Sketches: in two Parts. By *John Moore*, M. D. 8vo. *Strahan*, London, 1786.

7. Diſſertatio Medica Inauguralis de Tuſſi convulſiva. Auctore *Thoma Galley*, Anglo-Britanno. 8vo. Edin. 1785.

8. Diſputatio Phyſiologica Inaug. de Somno. Auctore *Joanne Hemming*, Britanno. 8vo. Edin. 1785.

9. Diſſ. Med. Inaug. de Leucorrhœa. Auct. *Carolo Johnſton*, Hiberno. 8vo. Edin. 1785.

10. Tentamen Medicum Inaugurale de quatuor vitæ gradibus. Auctore *Thoma Remington*, Britanno. 8vo. Edin. 1785.

11. Diſſ. Med. Inaug. de Dyſenteria. Auct. *Joanne Wilſon*, Anglo-Brit. 8vo. Edin. 1785.

12. Inſtrucion ſobre el mejor Metodo de Analizar las Aquas Minerales, y en lo poſible imitarlas. Por D. *Pedro Gutierrez Bueno*, Profeſſor de Farmacia, &c. 8vo. Madrid, 1785.

13. Analyſe Chimique et Concordance des trois Regnes. Par M. *Sage*. 8vo. Paris. 3 Tomes. 1786.

14. Ob-

14. Obſervations generales ſur les Maladies des Climats chauds, leurs Cauſes, leur Traitement, et les Moyens de les prevenir. Par M. *D'Azille*, Medecin du Roi à S. Domingue. 8vo. Paris, 1785.

15. Obſervations ſur les Obſtacles qui s'opposent aux progrès de l'Anatomie. Par M. *Tenon*, Profeſſeur Royal au College de Chirurgie, de l'Academie Royale des Sciences. 4to. Paris, 1785.

16. Enumeratio Lichenum iconibus et demonſtrationibus illuſtrata *G. Franc. Hoffmann*, Faſcicul. I. 4to. Erlangen, 1784. c. Tab. Æneis VIII.

17. *Caroli a Linné*, Equitis, Amœnitates Academicæ. Vol. VIII. cum Tabulis Æneis. 8vo. Erlangen, 1785.

18. De effectibus Terræ Motus in Corpore humano. Auctore *Vincent. Mignani*, M. D. 8vo. Bologna, 1784.

19. De Camphora et partibus quæ eam conſtituunt, Diſſertatio Inauguralis Chemica. Auctore *Dav. Aug. Joſua Frid. Koſegarten*, Sverino-Megapolitano. 4to. Goettingæ, 1785.

